# Spatial and temporal dispersion of ventricular repolarization in pediatric patients with congenital long QT syndrome

**DOI:** 10.1016/j.hrcr.2025.11.027

**Published:** 2025-12-09

**Authors:** Lixia Dai, Can Zhang, Nawin L. Ramdat Misier, Mathijs S. van Schie, Laura Bear, Michel Haissaguerre, Yannick J.H.J. Taverne, Natasja M.S. de Groot

**Affiliations:** 1 Department of Cardiology, Erasmus Medical Center, Rotterdam, The Netherlands; 2 Department of Cardiology, Bordeaux University Hospital (CHU), Pessac, France; 3 Department of Cardiothoracic Surgery, Erasmus Medical Center, Rotterdam, The Netherlands

**Keywords:** Long QT syndrome, Pediatric, Activation-recovery interval, Epicardial mapping, Ventricular tachyarrhythmias

**Key Teaching Points**

• Pediatric patients with congenital long QT syndrome (LQTS) already have a large spatial and temporal dispersion of ventricular repolarization, which cannot be assessed by standard surface electrocardiography.
• High-resolution epicardial mapping revealed a substantial spatial dispersion of the corrected activation-recovery interval (cARI) over a distance of 2 mm.
• Temporal dispersion of cARI over 100 ms occurred within only 5 seconds, reflecting marked beat-to-beat variability.
• Pronounced repolarization dispersions may underlie the increased susceptibility of young patients with LQTS to malignant ventricular tachyarrhythmias.

## Introduction

Congenital long QT syndrome (LQTS) is a genetic disorder characterized by prolonged corrected QT (QTc) intervals (Bazett formula) longer than 450 ms in male patients and 460 ms in female patients on the 12-lead surface electrocardiogram (ECG). This syndrome is caused by mutations in genes encoding ion channels, prolonging cardiac repolarization.^1^ Consequently, LQTS may give rise to life-threatening ventricular tachyarrhythmias and is a leading cause of sudden cardiac death (SCD) in young individuals without structural heart abnormalities.

The activation-recovery interval (ARI), an estimator of action potential duration, has been used to assess the duration and dispersion of ventricular repolarization.^2^, ^3^, ^4^ In patients with LQTS, there is, to our knowledge, only 1 study reporting on the spatial dispersion of ARI, which was measured by noninvasive mapping with ECG imaging.^5^ An accurate assessment of ARI is valuable for identifying patients at risk for SCD.

We report on inter- and intraventricular spatiotemporal dispersion of ARI, which is for the first time measured at a resolution of only 2 mm in 2 patients with LQTS who underwent surgical dual-chamber implantable cardioverter-defibrillator (ICD) implantation. This procedure provided the unique opportunity to perform epicardial mapping of the ventricles.

## Methods

The study was approved by the Erasmus Medical Center Medical Ethics Review Committee (MEC 2019-0543), and written informed consent was obtained from the legal guardians of both patients. This report complies with the principles of the Declaration of Helsinki.

Epicardial mapping of the ventricles was performed before surgical implantation of the dual-chamber ICD system. Standard general anesthesia was used, including midazolam, propofol, opioids (fentanyl/remifentanil), and rocuronium, along with routine adjuncts (eg, antibiotics, steroids, and analgesics). None of these drugs are known to significantly prolong ventricular repolarization.^6^ For this purpose, a custom-made unipolar electrode array (128 electrodes; diameter 0.45 mm; interelectrode distances 2 mm) was used, which covered an area of 32 × 16 mm.

Mapping was performed at 1 or 2 positions on the left ventricle (LV) and right ventricle (RV), with the electrode array parallel to the left anterior descending artery. At each mapping location, sinus rhythm recordings of either 5 seconds (case 1) or 10 seconds (case 2) were made.

The steepest negative slope of a ventricular unipolar *depolarization* potential was marked as the local activation time, and the steepest positive slope of a ventricular unipolar *repolarization* potential was marked as the local repolarization time (LRT). Electrograms with flattened T waves were excluded from the annotation. The time interval between the local activation time and the LRT is defined as ARI. The ARI was corrected for the mean cycle length by using the Bazett formula (cARI = ARI/ $\sqrt{mean\;cycle\;length}$), and the mean cARI was assessed for every electrode separately. The *maximum local dispersion* *of* *cARI* is defined as the maximum cARI differences between 2 adjacent electrodes, assessed in the horizontal and vertical directions at each electrode.

Temporal dispersion of cARI is measured as the standard deviation of the cARI (SD-cARI) measured at the same electrode across different heartbeats.

## Case report

### Case 1

**Figure 1:**
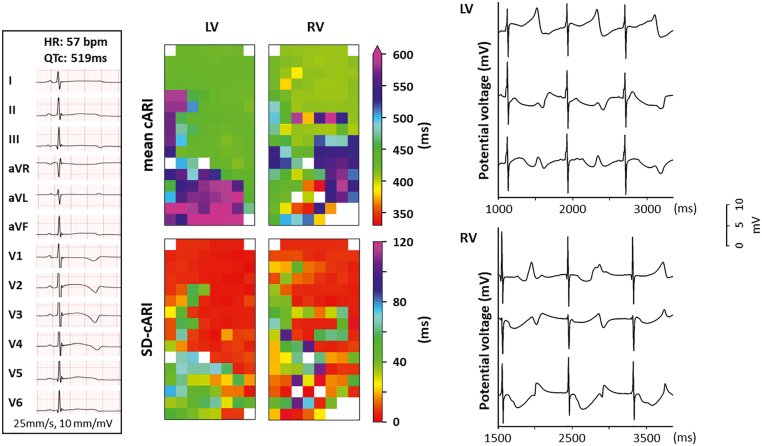
Case 1. *Left:* 12-lead surface electrocardiogram recorded before surgery, demonstrating prolonged QT intervals and abnormal T-wave morphology. *Upper middle:* Spatial distribution of mean cARI at the LV and RV. *Lower middle:* SD-cARI at the LV and RV. *Right:* Examples of unipolar potentials recorded from 3 recording sites during 3 consecutive beats at the LV and RV. cARI = corrective activation recovery interval; HR = heart rate; LV = left ventricle; QTc = corrected QT; RV = right ventricle; SD-cARI = standard deviation of the corrective activation recovery interval.

A 3-year-old girl presented with cyanosis, subsequently lost consciousness, and received basic life support. The ECG revealed a prolonged QT interval, with a QTc interval of 519 ms, as shown in the left panel of Figure 1. Subsequent DNA testing identified 2 compound heterozygous mutations in *KCNQ1* (c.569G>A, p.[Arg190Gln]) and *KCNH2* (c.1205A>G, p.[His402Arg]), respectively (LQTS type 1 [LQT1] and LQTS type 2 [LQT2]; Schwartz score 5). During treatment with propranolol and esmolol, continuous monitoring revealed frequent episodes of ventricular tachycardia (VT)/torsades de pointes. Given the recurrence of VT despite administration of antiarrhythmic drugs and the presence of compound mutations, implantation of a dual-chamber ICD was indicated. Before surgical lead implantation, epicardial mapping at the LV and RV could be performed at only 1 location per ventricle. This resulted in a total number of 744 LV potentials and 620 RV potentials, corresponding to 709 LV-LRT potentials (95%) and 540 RV-LRT potentials (87%).

The upper middle panel of Figure 1 shows color-coded repolarization maps visualizing the mean cARI measured during 5 seconds of sinus rhythm at every electrode at the LV and RV. These maps demonstrate a large spatial dispersion of mean cARI in both ventricles, ranging from 415 to 625 ms at the LV (mean 478 ms) and from 310 to 604 ms at the RV (mean 449 ms). The maximum local dispersion at the LV and RV was 76 ms (range, 3–210 ms) and 94 ms (range, 0–307 ms), respectively. The right panel shows examples of LV and RV unipolar potentials, demonstrating the beat-to-beat variation in T-wave morphology and consequently temporal variation in cARI. The spatiotemporal distribution in cARI at the LV and RV is visualized in the lower middle panel. At the LV, the mean SD-cARI was 21 ms, ranging from 2 to 76 ms; and at the RV, it was 24 ms, ranging from 0 to 106 ms.

During the follow-up period of 2 years, the patient has experienced repetitive appropriate ICD shocks, despite undergoing left stellectomy 1 year after ICD implantation.

### Case 2

**Figure 2:**
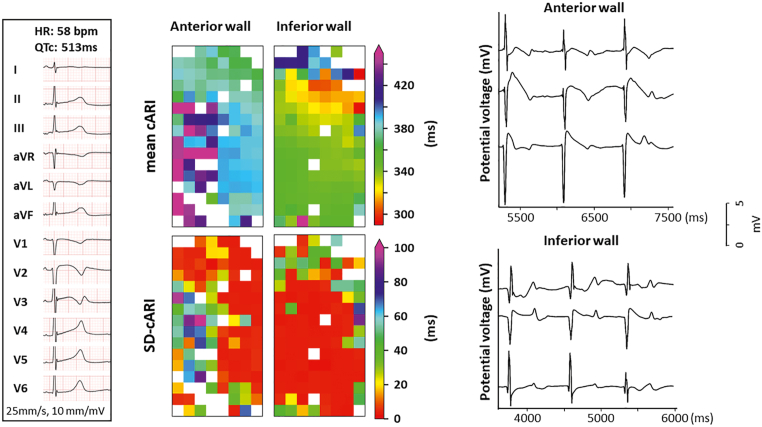
Case 2. *Left:* 12-lead surface electrocardiogram recorded before surgery, demonstrating prolonged QT intervals and abnormal T-wave morphology. *Upper middle:* Spatial distribution of mean cARI at the anterior and inferior walls at the RV. *Lower middle:* SD-cARI at the anterior and inferior walls at the RV. *Right:* Examples of unipolar potentials recorded from 3 recording sites during 3 consecutive beats at the anterior and inferior walls at the RV. Abbreviations as in Figure 1.

An 8-year-old girl was admitted with ventricular fibrillation and was successfully defibrillated to sinus rhythm. The ECG showed notched T waves in lead V_3_ and a prolonged QTc interval (QTc interval 513 ms), as shown in the left panel of Figure 2. A subsequent DNA test identified a mutation in *KCNH2* (LQT2; Schwartz score 4). Continuous rhythm monitoring during propranolol administration revealed frequent ventricular tachyarrhythmias and a QTc interval remaining longer than 500 ms. Consequently, a dual-chamber ICD was implanted. At the RV, epicardial mapping could be performed at 2 locations, but the LV could not be reached. A total of 1445 potentials were recorded from the RV anterior wall and 1452 potentials from the RV inferior wall, corresponding to 1042 LV-LRT potentials (72%) and 1299 RV-LRT potentials (89%).

The middle panel of Figure 2 shows the mean cARI and SD-cARI for the RV anterior and inferior walls, visualized in a similar way to Figure 1. The mean cARI at the RV anterior and inferior walls was 399 ms (range, 347–539 ms) and 347 ms (range, 293–473 ms), respectively. The maximum local dispersion at the RV anterior and inferior walls was 74 ms (range, 4–302 ms) and 58 ms (range, 3–226 ms), respectively, and the spatiotemporal variation in cARI was 23 ms (range, 2–147 ms) and 14 ms (range, 2–90 ms), respectively. The right panel of Figure 2 shows typical examples of RV anterior and inferior unipolar potentials, also demonstrating the beat-to-beat variation in T-wave morphology and consequently temporal variation in cARI. However, at both the anterior and inferior RV walls, there is also a change in the morphology of the preceding ventricular depolarization potential. During the follow-up period of 1 year, there were no ICD shocks.

## Discussion

By using an intraoperative, high-density, and high-resolution epicardial mapping approach, the spatiotemporal variation in refractoriness was measured in 2 pediatric patients with LQTS. In both patients, the longest cARI measured invasively was longer than the QTc interval measured on the surface ECG. In the patient with the compound mutation, the maximum local cARI dispersion reached 307 ms, with an overall spatial dispersion up to 294 ms.

Because of this invasive approach, there are no comparable data on cARI available from healthy pediatric subjects. Using noninvasive ECG imaging, Vijayakumar et al^5^ measured the cARI at the LV and RV in 7 healthy volunteers (mean age 28 ± 7 years) with structurally normal hearts and a cohort of patients with LQT1 (n = 9), LQT2 (n = 9), LQT3 (n = 5), and LQT5 (n = 2). The cARI of both ventricles measured in patients with LQTS (mean age 31 ± 16 years) was considerably longer (LQT1: 316 ± 28 ms; LQT2: 307 ± 36 ms; LQT3: 335 ± 18 ms; LQT5: 307 ± 36 ms) than in healthy subjects (288 ± 36 ms). Moreover, the maximum local repolarization dispersion of healthy volunteers was only 19 ± 13 ms, which was considerably shorter than in patients with LQTS (LQT1 99 ± 20 ms; LQT2 136 ± 36 ms; LQT3 110 ± 14 ms; LQT5 139 ± 28 ms).^5^

In these 2 pediatric patients with congenital LQTS, the mean cARI measured was longer (ranging from 347 to 478 ms), possibly reflecting the severity of the disease, as both patients demonstrated recurrent VT despite propranolol. In the first patient, the mean cARI at some recording sites was >100 ms longer than previously reported. Also, differences in mean cARI across only 2 mm were as high as 307 ms. This observation suggests that the compound mutations may cause a more severe cardiac channel dysfunction phenotype.^7^ Moreover, pediatric patients with LQTS are at significant risk for SCD. In a study involving children with LQTS, about 9% presented with cardiac arrest, and many others experienced syncopal events.^8^

Similarly, the larger dispersion may be explained by the severe phenotype. In addition, the “far-field” measures of ventricular refractoriness using the surface ECG are less accurate because of “averaging” of electrical activity. At all mapping sites in both patients, the longest cARI measured was longer (up to 625 ms) than the QTc interval measured on the surface ECG.

We also observed a beat-to-beat variation in T-wave morphology without changes in heart rate, sometimes related to beat-to-beat variation in activation potential morphology. Consequently, there was a *temporal* variation in cARI, which could be as high as 147 ms. Previous studies have demonstrated that T-wave morphology variation on the surface ECG is associated with cardiovascular events, such as life-threatening ventricular arrhythmias and SCD, even using a single-beat, single-lead ECG.^9^, ^10^, ^11^ Comparable to T-wave alternans, *temporal* variation in cARI may be an indicator of the increased risk of SCD. To our knowledge, this is the first study reporting on local temporal dispersion of ventricular repolarization in patients with LQTS.

## Conclusion

In these 2 pediatric patients with LQTS, localized areas of spatiotemporal dispersion of epicardial ventricular refractoriness were observed, which are larger than previously reported in adult patients with LQTS. These alterations may reflect the disease severity and could contribute to their presentation of malignant ventricular tachyarrhythmias. Further insights into the relation between genetic mutations, clinical profiles, and the various measures of refractoriness may increase our understanding of arrhythmogenesis underlying malignant ventricular tachyarrhythmias in young patients with LQTS.

## Disclosures

The authors have no conflicts of interest to disclose.
